# Identifying and understanding the contextual factors that shaped mid-implementation outcomes during the COVID-19 pandemic in organizations implementing mental health recovery innovations into services

**DOI:** 10.1186/s43058-021-00206-w

**Published:** 2021-09-15

**Authors:** Myra Piat, Megan Wainwright, Danielle Cherkas, Sébastien Leblanc, Eleni Sofouli, Marie-Pier Rivest, Hélène Albert, Regina Casey, Joseph J. O’Rourke, Lise Labonté

**Affiliations:** 1 Department of Psychiatry, McGill University and Douglas Mental Health University Institute, 6875, boul. LaSalle, Montréal, Québec, H4H 1R3 Canada; 2 Department of Anthropology, Durham University, Dawson Building, South Road, Durham, DH1 3LE UK; 3 Factor-Inwentash Faculty of Social Work, University of Toronto, 246 Bloor St W, Toronto, Ontario M5S 1V4 Canada; 4 École de travail social, Université de Moncton, 18, avenue Antonine-Maillet, Moncton, Nouveau-Brunswick E1A 3E9 Canada; 5 Department of Psychiatry, McGill University, Ludmer Research & Training Building, 1033 Avenue des Pins, Montréal, QC, H3A 1A1 Canada; 6 École de travail social, Université de Moncton, 18, avenue Antonine-Maillet, Moncton, Nouveau-Brunswick E1A 3E9 Canada; 7 École de travail social, Université de Moncton, 18, avenue Antonine-Maillet, Moncton, Nouveau-Brunswick E1A 3E9 Canada; 8 Department of Occupational Science and Occupational Therapy, Faculty of Medicine, The University of British Columbia, T-325, 2211 Westbrook Mall, Vancouver, British Columbia V6T 2B5I Canada; 9 Douglas Mental Health University Institute, 6875, boul. LaSalle, Montréal, Québec, H4H 1R3 Canada

**Keywords:** Canada, Consolidated Framework for Implementation Research, COVID-19, Guidelines, Recovery-oriented services, Mental health recovery, Mid-implementation, Outer setting, Pandemic, Supported housing

## Abstract

**Background:**

Seven housing and health services organizations were guided through a process of translating Chapter Six of the Canadian Guidelines for Recovery-Oriented Practice into a recovery-oriented innovation and plan for its implementation. At the time of the COVID-19 outbreak and lockdown measures, six of the seven organizations had begun implementing their chosen innovation (peer workers, wellness recovery action planning facilitator training, staff training and a family support group). This mid-implementation study used the Consolidated Framework for Implementation Research (CFIR) to identify contextual factors that influenced organizations to continue or postpone implementation of recovery-oriented innovations in the early months of the COVID-19 pandemic.

**Methods:**

Twenty-seven semi-structured 45-min interviews were conducted between May and June 2020 (21 implementation team members and six providers of the innovation (trainers, facilitators, peer workers). Interview guides and analysis were based on the CFIR. Content analysis combined deductive and inductive approaches. Summaries of coded data were given ratings based on strength and valence of the construct’s impact on implementation. Ratings were visualized by mid-implementation outcome and recovery innovation to identify constructs which appear to distinguish between sites with a more or less favorable mid-implementation outcomes.

**Results:**

Four mid-implementation outcomes were observed at this snapshot in time (from most to least positive): continued implementation with adaptation (one site), postponement with adaptation and estimated relaunch date (four sites), indefinite postponement with no decision on relaunch date (one site), and no implementation of innovation yet (one site). Two constructs had either a negative influence (external policies and incentives—renamed COVID-19-related external policy for this study) or a positive influence (leadership engagement), regardless of implementation outcome. Four factors appeared to distinguish between more or less positive mid-implementation outcome: adaptability, implementation climate and relative priority, available resources, and formally appointed internal implementation leaders (renamed “engaging implementation teams during the COVID-19 pandemic” for this study).

**Conclusions:**

The COVID-19 pandemic is an unprecedented outer setting factor. Studies that use the CFIR at the mid-implementation stage are rare, as are studies focusing on the outer setting. Through robust qualitative analysis, we identify the key factors that shaped the course of implementation of recovery innovations over this turbulent time.

**Supplementary Information:**

The online version contains supplementary material available at 10.1186/s43058-021-00206-w.

Contributions to the literature

- The outer setting is the least studied domain of the consolidated framework for implementation research (CFIR). The COVID-19 pandemic is an unprecedented outer setting factor. This study contributes to building the evidence base around the impact of this outer setting factor on implementation.
- Unlike most implementation studies that use the CFIR for post-implementation data analysis, we used the CFIR to design interview guides and undertake qualitative analysis at the mid-implementation phase.
- Our research identifies CFIR constructs that appeared to be most associated with continued implementation of adapted recovery-oriented innovations into services in the early months of the COVID-19 pandemic.

## Background

### Implementing mental health recovery into services

Recovery is defined as living a satisfying, hopeful, contributing life despite the limitations of mental illness [1]. In services that are recovery-oriented, people living with serious mental illness are supported “to define their own needs, goals, dreams, and plans for the future” (p.1474) [2]. Recovery-oriented services demonstrate a focus on connectedness, hope and optimism about the future, identity, meaning in life, and empowerment [3]. Achievement of this requires the recovery-transformation of services traditionally focused on symptom reduction, clinical outcomes, and professional dominance and control over the “expertise of lived experience” [4–10].

As the dominant paradigm in mental health, recovery is the focus of national mental health plans in G8 countries including Canada’s 2012 mental health strategy, *Changing Directions, Changing Lives* [11], and provincial policies [12–16]. Implementing recovery guidelines is therefore closely tied to achieving these policies and strategies. The Mental Health Commission of Canada launched the *Guidelines for Recovery Oriented Practice* [17] in May 2015 (herewith referred to as the Recovery Guidelines). These guidelines are based on a review of the literature, other international guidelines [18, 19], best practices, and a nation-wide consultation. They include six chapters on different dimensions of recovery-oriented practice including Chapter Six which focuses on system and service-level transformation. Although each chapter includes a list of possible actions managers and decision makers can take, guidance for how to actually implement specific interventions that meet the goals of each guideline is lacking.

In 2017, we received funding from the Canadian Institutes of Health Research for a 5-year project entitled *Implementing Mental Health Recovery Guidelines into Services: A Pan Canadian Study* [20]. The aim is to translate Chapter Six of the Recovery Guidelines into tangible innovations to be implemented into seven housing and health service organizations in five Canadian provinces. A detailed description of the study and its implementation strategies are published elsewhere [Piat et al.: Translating mental health recovery guidelines into recovery-oriented innovations: A strategy combining implementation teams and a facilitated planning process, under review]. The primary implementation strategy used was establishing implementation teams at each of the seven sites [21–23]. Implementation Teams were made-up of diverse stakeholder groups (tenants, service providers, managers, family members, knowledge users) who took on the role of formally-appointed implementation leaders [24]. Implementation teams were guided by the researchers through a 12-meeting planning process and subsequent bi-monthly and monthly consultation and coaching meetings [Piat et al.: Translating mental health recovery guidelines into recovery-oriented innovations: A strategy combining implementation teams and a facilitated planning process, under review]. The fact that sites were not told what to implement by the research team is a unique feature of this project. Rather, researchers facilitated a process whereby each implementation team chose a sub-guideline of Chapter Six to focus on, chose an evidence-based innovation based on their needs and resources, and planned for its implementation. Planning included a systematic approach to identifying barriers and strategies [25] and creating an implementation plan.

### The COVID-19 pandemic as an outer setting factor

The Consolidated Framework for Implementation Research (CFIR) [24] is a compilation of factors known to influence implementation of new innovations into organizations. It includes 39 constructs (contextual factors) grouped into five domains: characteristics of the intervention, outer setting, inner setting, characteristics of individuals, and process. The least elaborated and researched of the five CFIR domains is the outer setting [26–31] which includes four CFIR constructs: patient needs and resources, cosmopolitanism, peer pressure and external policies and incentives. Outer setting is defined as the wider economic, social, cultural, and political context outside the organization and in which it is situated [24]. The paucity of research on outer setting factors has been explained in part by the lack of quantitative measurements due to difficulty operationalizing and isolating these as a unit of measurement [30]. However, qualitative research methods are also appropriate for understanding how outer setting factors shape implementation [32].

Recent research using CFIR and studying the outer setting explicitly has helped to broaden the scope and specificity of this domain. Researchers have suggested additional constructs like coercive, mimetic, normative pressure, uncertainty in the task environment, transaction costs [28], and community characteristics [33]. Others highlight the importance of cultural adaptation of innovations and community networks [34], and the association between implementation funding and per capita income, political party in power and health system [27]. Noting that the outer setting can include everything from community-level to national-level contextual factors, the scoping review by Nilsen and Bernhardsson [31] categorized contextual factors into micro, meso, and macro levels. The macro level was defined as “even broader,” “outside,” and “influences from the wider environment” (p.13). Rogers et al. [35] propose the umbrella term “system-level determinants” to encompass social, political and economic environments. We would add that outer setting factors are not just governmental or national in level, but international and even global. The outbreak of the COVID-19 pandemic for instance was an undeniable global macro level outer setting factor that impacted implementation in our study. As argued by Becker et al. [26] “COVID-19 underscores the importance of the outer setting, and offers an unprecedented window through which to view the interacting effects of the inner and outer settings” (p.2). While the imperative to implement policies around staffing, movement, face-to-face services, and social distancing originated outside the organizations involved, these policies had to be translated and enacted within the inner setting.

### Mid-implementation outcomes during the COVID-19 pandemic

Kirk et al. [36]’s review of the use of CFIR in research reported that in the majority of cases CFIR is used at the post-implementation phase for data analysis only. They recommended that researchers integrate the CFIR into the whole research process. In our study, the CFIR was transformed into the CFIR Card Game [25] to work with implementation teams in the planning stage, and CFIR was also used to design interview questions and guide analysis at pre-, mid-, and post-implementation phases of the project. This article reports on the analysis of mid-implementation data. Similar to post-implementation studies that seek to use CFIR to understand what the drivers of implementation success or failure were, we sought to understand what factors might be shaping mid-implementation outcomes.

In the face of an unforeseen outer setting factor like a global pandemic, whether or not organizations continued to adopt an innovation (adoption), as well as whether they could or could not continue as intended (fidelity), are key early to mid-implementation outcomes [37]. While an event like the COVID-19 pandemic can fast-track implementation by removing previously prohibitive barriers [26, 38], and push previously reluctant decision makers to take radical decisions [39], it can also bring innovations predicated upon face-to-face interaction and engagement to a sudden halt. Adaptation, which some argue is itself an implementation outcome [32], was the only route to continued adoption in our study. Going virtual because of COVID-19 is an example of reactive adaptation [40] but is an entirely different reason for adaptation than previously described [41].

Authors have begun describing the impact the COVID-19 pandemic has had on their projects and how organizations have or have not adapted [26, 42, 43]. Becker et al. [26] describe how one of three outcomes was observed among Opioid Treatment Programs implementing contingency planning as a result of the COVID-19 pandemic—discontinuation with no plan for resumption, pause and develop plans to resume services once social distancing guidelines were relaxed, and adaptation to an online version without service disruption. In our study, we categorized mid-implementation outcomes similarly (see the “Results” section). While these recent contributions help to conceptualize mid-implementation outcomes in the context of the COVID-19 pandemic, research is needed to study the factors that drove these outcomes. To the best of our knowledge, we are the first to do this in the context of the COVID-19 pandemic and the implementation of recovery into services.

### COVID-19 response in Canada

Canada’s ten provinces and three territories take responsibility for their provincial health systems. Shortly after the Director-General of World Health Organization, Dr Tedros Adhanom Ghebreyesus, declared the COVID-19 outbreak as a pandemic on March 11, 2020 [44], each provincial health authority decided on the required strategies and actions to minimize the transmission of the virus, address the financial impact of the pandemic, and ensure the safety of its population. Provincial authorities in Québec [45], Ontario [46], British Columbia [47], Manitoba [48], and New Brunswick [49] where the participating organizations in this study were located declared a state of emergency as the number of new COVID-19 cases among the general population, and especially in long-term facilities for older adults, increased steeply. In addition to policies restricting movement, closing schools, and restricting social gatherings in public and private spaces [50], persons living in supported housing facilities for people living with a mental illness were under even stricter lockdown policies in some provinces, for example, being banned from having any visitors at all, to being unallowed to leave the premises unaccompanied even if only for essential purchases.

## Methods

### Study aim and design

The aim of the current qualitative study was to identify contextual factors that influenced if and how organizations continued implementing their innovations in the first 2 to 3 months of the COVID-19 pandemic in Canada. We applied the Standards for Reporting Implementation Studies (StaRI) [51] and the Consolidated Criteria for Reporting Qualitative Studies (COREQ) [52] checklists as reporting guides for this manuscript (Additional files 1 and 2).

### Settings and innovations

The research was conducted in seven organizations across five Canadian provinces participating in the project: two publicly funded organizations (Québec: an integrated university health and social service center, and New Brunswick 1: a community health centre), and five not-for-profit organizations (Ontario, British Columbia, New Brunswick 2, Manitoba 1 and Manitoba 2). Six provide housing services for adults with mental health problems, and one provides mental health services (New Brunswick 1). The introduction of this paper described the process of working with implementation teams to select an innovation and plan for its implementation. Innovations selected included staff training programs in mental health recovery [17] (Manitoba 2, Ontario, New Brunswick 2), hiring peer workers [53] (Manitoba 1, Québec), Wellness Recovery Action Planning (WRAP) training for people with lived experience and staff to become WRAP facilitators [54] (British Columbia), and a family support group program [55] (New Brunswick 1). How these sites were selected is described in detail elsewhere [Piat et al.: Translating mental health recovery guidelines into recovery-oriented innovations: A strategy combining implementation teams and a facilitated planning process, under review].

### Participants and recruitment

We purposefully recruited (via email or telephone) three to five participants per research site from the implementation team and providers of the innovation (newly hired or contracted trainer, facilitator or peer worker) for a total of 27 participants (Table 1). Our sampling was guided by the concept of information power [56]. The information power concept posits that the more relevant information a sample holds, the fewer participants are needed. In our case the fact that the aim of the study was narrow, our sample highly specific (implementation team members and providers of the innovation), our study and data collection informed by established implementation theory (CFIR), and that there was pre-existing strong communication between the research team and the participants meant that a smaller sample size per site sufficed [56]. We identified the formal or informal leader of each implementation team and prioritized their recruitment. We successfully recruited all but two of our planned purposive sample. A service provider was unavailable during the recruitment period and was replaced by another implementation team member, and a service user was unreachable and unable to be replaced.

**Table 1:** Study participant characteristics by site and stakeholder group

Sites	Stakeholder groups				Totals (***N***=27)
	Service users on IT (*n*=6)	Service providers on IT (*n*=7)	Managers on IT (*n*=8)	Providers of the innovation (*n*=6)	
Québec	1	1	2**	1	5
Manitoba 1	1	1	1	1	4
New Brunswick 1	1*	1	1	1	4
Ontario	1	1	1	1	4
Manitoba 2	–	1	1	1	3
New Brunswick 2	1	1	1	–	3
British Columbia	1	1	1	1	4
**Age in years M(SD)**	64 (8.4)	44.7 (17.8)	45.5 (11.04)	49.6 (9.8)	50.37 (14.08)
**Gender**	4(F), 2(M)	4(F), 3(M)	7(F), 1(M)	4(F), 2(M)	19(F), 8(M)

*IT* implementation team, *M* mean, *SD* standard deviation, *F* female, *M* male. *This is a family member of a service user. **This includes one housing proprietor

### Data collection

Twenty-seven online semi-structured interviews lasting approximately 45 min were conducted between May and June 2020 using a range of web-based videoconferencing software. We kept interviews to strictly 45 min out of respect for participants who were under enormous pressure at this stage of the pandemic. The aim was to use audio and video (webcam) for all interviews, but due to internet or hardware issues, seven interviews were audio-only (no video). Twenty-one were with members of the implementation teams in all seven sites (five service users, one family member, seven service providers, one housing proprietor, and seven managers). Housing proprietors is a stakeholder group specific to Québec where housing consists of privately owned residences and their owners referred to as housing proprietors. The remaining six were with providers of the innovation (three externally contracted trainers, two newly hired peer workers, and one support group facilitator).

One interview guide was used for all interviews and was based on the CFIR using as a starting point the Interview Guide Tool available at www.cfirguide.org (Additional file 1). Three members of the national research team (MP, MW, ES) met to prioritize domains and constructs, basing their decisions on anecdotal knowledge of context and the kinds of issues organizations participating in the research were facing (e.g., considering online options, imperative to implement new COVID-19 policies). Questions targeted COVID-19 and related policies (outer setting), adaptability of the innovations (characteristics of the intervention), implementation climate, relative priority, leadership engagement and available resources (inner setting), and the work of the implementation team (process) (see Additional file 3 for guide). Despite not explicitly probing for more than seven CFIR constructs due to time-constraints, questions were broad and open-ended enough to allow participants to speak to other constructs (which they did as evidenced by having at least some data coded to 31 of the 39 constructs).

Nine researchers (7 female, 2 male) conducted the interviews (PhDs: MP, MW, M-PR, RC, HA, PhD student: ES, Masters students and research assistants: SL, JO, LL). Two Masters of Social Work practicum students (including DC) sat-in on almost all of the interviews to take notes as a back-up to the voice recording. Each of the Co-Investigators (RC, M-PR, HA) were the lead researchers for sites in their home province. The Principal Investigator (MP) was the lead researcher in four sites in three provinces. Two sites (Québec and Richibucto 1) were within a large provincial government health centre or network that had affiliations with the universities of the lead researchers (MP, M-PR). Only the PI (MP) was an employee of the health centre. However, her employment was with a research institute—a separate department from the housing services that participated in the study. For mid-implementation data collection, because interviews were online and did not require travel, participants were interviewed by researchers from a different site. [Piat et al.: Researchers’ experiences of switching research sites for online interviews during the COVID-19 pandemic: a Research Note. forthcoming].

### Data analysis

All interviews were audio-recorded and transcribed verbatim. We employed the approach described in Damschroder and Lowery [57] and detailed on https://cfirguide.org/evaluation-design/qualitative-data/. This method has been used extensively with qualitative data to identify factors associated with implementation outcomes [58–62]. We followed three steps in analysis: coding data to CFIR constructs, rating constructs, and qualitative interpretation of patterns in the data.

#### Coding data to CFIR constructs

We performed a qualitative content analysis by deductively coding transcripts to the 39 CFIR constructs using the software program NVivo 12 [63]. Data that did not fit the existing constructs were inductively coded to new categories. Each transcript was independently coded by two of three researchers (MW, DC, SL) who met weekly to compare coding and resolve differences through consensus discussion before moving on to the next transcript. Coding was discussed with two other researchers at regular intervals (MP, ES). We also inductively combined or renamed codes to better reflect our data. We originally had the construct “implementation climate” and its sub-construct “relative priority” as separate codes since one question in the guide targeted the general climate (how receptive people are), and another relative priority (how important is it compared to other priorities). However, participants’ responses blurred the two and after recognizing that we were frequently coding data chunks to both, we combined them into one. We also re-named the construct “external policy and incentives” to “COVID-19-related external policy” and the sub-construct “formally-appointed internal implementation leaders” to “engaging implementation teams during the COVID-19 pandemic”, to better reflect the data. Two additional outer setting constructs were created through inductive coding “Local severity of the COVID-19 pandemic and quality of response” and “Priority given to mental health in wider society”. Coders could not be blinded to the outcomes as was done by Damschroder and Lowery [57] since the mid-implementation outcome in this case was made obvious in the interview itself.

#### Rating constructs

“Case memos” which include a summary, a rating, and a rationale [57] were created through combined use of NVivo12 and Excel. For summaries, a Framework Matrix table was created in NVivo12 for each site. This enabled the researchers (MW, DC, SL) to write a summary for each construct while viewing the interview data coded to that construct. For ratings and rationales, the seven matrices (one for each site) were exported to Excel. We used the Rating Criteria provided in Table 2 of Damschroder and Lowery [57] and the Rating Rules on www.cfirguide.org as our guides to rating. Two researchers independently assigned a rating to each construct based on valence (positive (+) or negative (−) impact on implementation) and strength (weak (1) or strong (2) impact on implementation) and wrote a rationale. Ratings ranged from −2, −1, 0, +1, +2. 0 reflected a neutral or mixed influence and a * sign identified the existence of a view contrary to the overall rating. Ratings and rationales were compared and a final rating and rationale reached through consensus discussion.

**Table 2:**
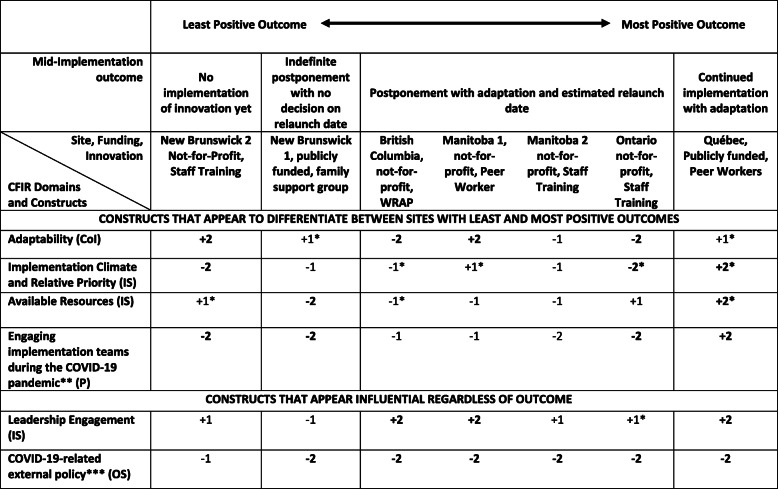
Ratings assigned to CFIR constructs by mid-implementation outcome

*Indicates that there exists a view that is contrary to the overall rating. **Formally appointed internal implementation leader construct. *CFIR* Consolidated Framework for Implementation Research, *CoI* characteristics of the Intervention domain, *OS* outer setting domain, *IN* inner setting domain, P process domain. ***External policies and incentives construct

#### Qualitative interpretation of patterns in the data

The online CFIR Guide describes four possible approaches for interpreting qualitative data that have been coded to CFIR constructs and rated: qualitative analysis, correlation analyses, qualitative comparative analysis (QCA), and statistical or simulation models. The goal of each is to “identify constructs that appear to distinguish between organizations with high and low implementation success” [64]. We used the qualitative analysis approach, as in Damschroder and Lowery [57], because it is the most appropriate for a study with a small number of cases and participants, and our expertise is in qualitative research methods. QCA for example, requires a minimum of 10 cases [65]. In the qualitative analysis approach, patterns are identified by using a matrix (template provided on CFIR Guide site) to sort sites by implementation outcome (Tables 2 and 3). The researchers interpret the data in the matrix in order to identify compelling patterns. Since our study is a mid-implementation study, rather than a post-implementation study, our outcomes were not framed in terms of high or low success, but rather by different degrees of adoption of the innovation in the organization at the time of the COVID-19 outbreak. The mid-implementation outcomes can be seen in Table 2 and are described in the findings section.

**Table 3:** Ratings assigned to CFIR constructs by innovation being implemented

Innovation	Family support group	WRAP	Staff training			Peer workers	
CFIR constructs\ Site	New Brunswick 1 publicly funded, family support group	British Columbia not-for-profit, WRAP	New Brunswick 2 not-for-profit, staff training	Ontario not-for-profit, staff training	Manitoba 2 not-for-profit, staff training	Manitoba 1 not-for-profit, Peer worker	Québec publicly funded, peer workers
Adaptability (CoI)	+1*	**−2**	**+2**	**−2**	**−**1	**+2**	+1*
COVID-19-related external policy (OS)	**−2**	**−2**	**−**1	**−2**	**−2**	**−2**	**−2**
Implementation climate and relative priority (IS)	**−**1	**−**1*	**−2**	−2*	**−**1	+1*	+2*
Leadership engagement (IS)	**−**1	**+2**	+1	+1*	+1	**+2**	**+2**
Available resources (IS)	**−2**	**−**1*	+1*	+1	**−**1	**−**1	+2*
Engaging implementation teams during the COVID-19 pandemic (P)	**−2**	**−**1	**−2**	**−2**	**−**2	**−**1	**+2**

* Indicates that there exists a view that is contrary to the overall rating. *CFIR* Consolidated Framework for Implementation Research, *WRAP* Wellness Recovery Action Planning. *CoI* characteristics of the intervention domain, *OS* outer setting domain, *IN* inner setting domain, *P* process domain

Two analysis and interpretation meetings were held (MW, DC, SL, MP, ES). Meeting 1 focused on jointly reviewing Table 2 to collectively identify and discuss emergent patterns. Our focus was on constructs which appeared to distinguish between sites with more or less positive outcomes. We bolded and highlighted positive ratings in green and negative ratings in red to make it easier to see patterns. It is important to note that the ratings in the table are not the final results of the analysis and cannot be considered in isolation from the “Results” section of this manuscript. Rather, they are the starting point for a qualitative interpretation of the data. In the first instance, we looked for whether some constructs unambiguously distinguished between sites—for example, all negative ratings for sites with less positive outcomes, and all positive ratings for sites with more positive outcomes. This was the case for only one construct. However, because our data was complex on a number of levels—multiple innovations being implemented, multiple stakeholder group perspectives, and mid-implementation outcomes—we used a more qualitative approach to identifying possible patterns in the data. For example, were *most* ratings on the more positive side of the spectrum positive or vis-versa? Were there strongly positive or strongly negative ratings at *either end* of the spectrum? We also considered a pattern to exist even when a disconfirming case was present (e.g., a positive rating in a site with a less positive outcome) if this disconfirming case could be explained by the qualitative data. We also brought our in-depth knowledge of the sites to bear in our interpretation of the data. We hypothesized at the first data interpretation meeting that some variations in the data, that is outliers or disconfirming cases, might be explained by the type of innovation being implemented. We therefore spent a second data interpretation meeting discussing the matrix organized by innovation (Table 3), which helped broaden our interpretation of the outcome data patterns. In writing-up the findings, we drew on the summaries and underlying quotes and examples in the NVivo12 project to illustrate how the construct appeared to shape mid-implementation outcomes.

## Results

At the time of the pandemic outbreak, six of the seven sites had started implementing their chosen innovations. New Brunswick 2 was an outlier because it was the only site to have not yet started implementation but was on the cusp of doing so. We determined there to be four mid-implementation outcomes. We present results per site, running from most positive (continued implementation with adaptation) to least positive outcomes (Postponement with adaptation and estimated relaunch date, Indefinite postponement with no decision on relaunch date, and No implementation of innovation yet).

Our analysis matrices (see Additional files 4 and 5 for matrices including all constructs) show that there is variation in the data, rather than a perfect pattern for each construct of all positive ratings for more positive outcomes, and all negative ratings for less positive outcomes. To make sense of these variations, the qualitative data is imperative; therefore, we do not recommend looking at the matrices in isolation or taking them as the results of the analysis on their own. Through our collaborative approach to analysis, we have identified four constructs that appear to be influential on mid-implementation outcomes: adaptability, implementation climate and relative priority, available resources, and engaging implementation teams during the COVID-19 pandemic (see Table 2). Before presenting data site-by-site to illustrate how these constructs influenced mid-implementation outcomes, we will explain our interpretation of the key constructs in general. Overall, we considered it telling that the only site to have continued implementation with adaptation was also the only site to have positive ratings for all four of these key constructs. If we ignore some of the disconfirming data that New Brunswick 2 introduces (we explain these below), patterns become clearer. First of all, engaging implementation teams during the COVID-19 pandemic was a positive influence only in the site that continued implementation with adaptation. Secondly, available resources were predominantly a negative influence on implementation except for the one site that continued implementation with adaptation where it was a strongly positive influence. Thirdly, implementation climate was strongly positive only in the site that continued implementing with adaptation, and we believe the fact that the innovation was peer workers was important in this case and explains why the only other site that implemented this innovation also had a positive rating (Manitoba 1). Fourthly, peer work was considered especially adaptable thus explaining the positive influence of adaptability in both the site with the most positive mid-implementation climate, and one site with the next best outcome (Manitoba 1). The fact that the family support group has elements in common with peer support might explain why adaptability was also a weakly positive influence in New Brunswick 1. Innovations that were based on training were considered the least adaptable and this perception was an important driver for the decision not to continue with adaptation at this snapshot in time.

Two other constructs did not differentiate between less and more positive implementation outcome but rather had a unanimous, or almost unanimous positive or negative effect on implementation. COVID-19-related external policy had a negative influence on implementation across all sites as the policies prohibited the innovations from continuing as planned. Leadership engagement had a positive influence across six of the seven sites. In the following, we illustrate the impact of the key constructs in each site by implementation outcome.

### Continued implementation with adaptation

#### Québec—peer workers

Québec was the only site to have been able to continue implementing their innovation without interruption by adapting it to a virtual format. We considered this the most positive mid-implementation outcome among all sites. At the time of data-collection, two peer workers had been hired (February 2020) to provide peer support in three supported housing sites and had been working for approximately 1 month. Lockdown policies in Québec were especially strict for supported housing as it was categorized similarly to health services in general. Face-to-face services were permitted only for what was deemed “essential services” which in the case of mental health services, was primarily the provision of medication and testing and screening. Teleworking policies also led to peer workers having to stay home and work remotely. In response, the implementation team, along with the newly hired peer workers, adapted the innovation by moving forward with virtual group and one-on-one peer support using web-based teleconferencing tools (tablets, computers, webcams). Notably, it was the only site to have strongly positive ratings for three influential constructs: implementation climate and relative priority, available resources, and engaging implementation teams.

Interviewees noted that the implementation climate was positive for virtual adaptation because there was a strong perception that peer support was an even greater priority during COVID-19 since service users were strongly impacted by lockdown policies.

It’s that our residents are very isolated, they are very very isolated in normal times, so in the time of COVID, they are also isolated, family members aren’t always involved, they are, it’s sad to say, but sometimes they are used to being alone, or the people in their network are the people they live with, so having peer workers is an additional tool. (Manager, Québec)

They also noted how the resources needed were made available with little delay—this included the purchase of a tablet for each of the housing sites, available or upgraded Internet, and peer workers with access to the needed devices from home. Leaders’ support was important for facilitating access to needed resources. Adaptability was rated as weakly positive, because though yes they thought peer support could be adapted to a virtual format, it was seen as second best to in-person. “The thing that I have to say is it’s better in person” (Tenant, Québec). Furthermore, some noted that although tablets were made available, they did not provide the sound and video quality needed for an optimal adaptation.

They had the four participants sit a bit far from the tablet so that we could see all of them on the screen, but with that the sound becomes difficult, because they are talking from far away, sometimes we have to ask them to repeat themselves, it doesn’t bother them to, but it makes the connection a bit more distant. (Peer support worker, Québec)

The implementation team played an important role in planning for the adaption of the innovation, and incidentally, the Québec implementation team was the only team that continued meeting uninterrupted throughout the pandemic.

… the implementation team, whether it was myself or others, decided or really said “no, no, we have to continue, we have to put it in place, we have to ensure that it keeps going”, that allowed for it to be maintained, for it to continue. (Service Provider, Québec)

Probably if the committee [implementation team] had only existed for a month, we might have said to ourselves, ok, let’s pick this up later, but you know a year of working on this, for sure they don’t want to put it on ice and they will find a way of maintaining meetings and continuing to be involved despite the adverse context. (Manager, Québec)

### Postponement with adaptation and estimated relaunch date

Four sites had postponed implementation at the time of data collection, but reported having an estimated date for relaunching an adapted innovation. We considered this the second most positive mid-implementation outcome. For the four key constructs identified in our analysis, all were negatively rated except two constructs for Manitoba 1 and one for Ontario.

#### British Columbia—Wellness Recovery Action Planning

In British Columbia, Part 1 of the WRAP facilitator training program was completed in December 2019. Part 2 of the training had been due to commence in April 2020 but was postponed. British Columbia had negative ratings for all of the key constructs. When in-person activities were banned due to COVID-19, the team considered adapting the innovation to an online format but came to the conclusion that WRAP as an innovation was not conducive to an online format:

Doing WRAP is too personal to do it over a computer and then to leave your feelings by yourself. We’ve decided that’s not a safe or healthy thing to do. (service user, British Columbia)

In terms of implementation climate and relative priority, although all members of the IT team perceived WRAP as a useful tool for managing reactions to and the impact of COVID-19 for both staff and tenants, COVID-19 resulted in a de-prioritization of WRAP training by staff members who were more focused on controlling infection, adhering to protocols and managing increased stress. Implementation team meetings were also temporarily put on hold during the early outbreak, with team members lacking resources to meet online, and being pulled towards other priorities.

The early days of the outbreak I think there was a lot of panic, and I think just in general there wasn’t the bandwidth to be able to deal with continuing on with the implementation team. (Manager, British Columbia)

Although some resources were secured (e.g., financing for the innovation), and new resources available (venues that could allow socially-distanced training in due course), there was an overall deficit in terms of human resources, that is staff time, for continuing with the planned implementation of part 2 of the WRAP program.

#### Manitoba 2—staff training

This site chose staff training in recovery and had contracted an external trainer to provide 12 in-person training sessions to staff as well as interested tenants. At the time of postponement, two of 12 sessions had been completed. This site also had negative ratings for all key constructs. Although the trainer described how the organization was offered an online alternative either through real-time Zoom sessions or pre-recorded online workshops, the implementation team, in consultation with staff in the organization, chose instead to postpone until July when they expected in-person trainings would be permitted with social distancing.

… you could do online, but something like this where I think that kind of the value of being able to discuss things as a group really adds a richness that we didn’t want to lose… (Manager, Manitoba 2)

Implementation team planning meetings were put on hold due to limited technological resources at the housing site in the early days of the outbreak. Also, members of the implementation team, as well as members of staff who were supposed to participate in the training, were overwhelmed with additional roles and tasks:

…half of the time that our case coordinators would have had for things like meetings and, you know, training and stuff, that time was just reassigned to cleaning. (Service provider, Manitoba 2)

#### Ontario—staff training

Three of 10 staff recovery training sessions had been completed with an external trainer at the time when it was decided the training should be postponed due to a ban on in-person meetings. Like other sites, COVID-19 policies had an immediate negative impact:

Ok, so in March we got the, the kind of the 1st kind of “there’s something wrong” and the moment we got the memo from the organization and the province says no more than 5 people in a room, everything stopped, and it’s stalled. (Manager, Ontario)

Three of the four key constructs were rated negatively, and one positively. In contrast however to other sites with the same outcome, available resources were rated as a weakly positive influence on implementation. This was because this was the only site with this implementation outcome that attested to having the technological resources to conduct training online. However, the driving force behind choosing not to go virtual and instead postpone was a negative implementation climate and lowered priority of training in the face of COVID-19 among staff who were the primary target group for training. Participants explained the additional tasks staff were having to take on, and in the words of a tenant:

They [staff] don’t, they don’t seem to be receptive right now, I gather from just a few comments that some staff members that they decided among themselves that this, this project is on the backburner as far as they are concerned. (Tenant, Ontario)

Overall, although they thought that the training was technically adaptable to an online format, they believed doing so would have too many negatives, including a lack of human connection, interaction, and being self-conscious about being on camera. In terms of the implementation team’s continued engagement with planning, meetings were suspended during the early days of the outbreak, but resumed virtually a few weeks prior to the interview. However, there were technological challenges, including a lack of access to needed hardware and software for two members of the team. Furthermore, absences at meetings demonstrated a change in relative priority of implementation team work compared to other priorities.

#### Manitoba 1—peer workers

A peer worker had been hired and was in the first month of delivering peer support when they had to take leave in accordance with COVID-19 policies. We can observe that Manitoba 1, unlike the other three sites with the same mid-implementation outcome, had positive ratings for adaptability and implementation climate and relative priority, and this relates specifically to the innovation chosen. Like Québec, they considered the peer support role to be adaptable and an even greater priority due to COVID-19. They had plans for adapting the peer worker role, for example by the peer worker making telephone call check-ins to residents, helping with food delivery, and, once allowed, having socially-distanced conversations with tenants on an outdoor patio.

When [peer worker] does come back, we’re still going to utilize him because the intent of his job is actually more important now because people are feeling more isolated. (Manager, Manitoba 1)

Resources were not a strong barrier because budgets for the peer worker were secured. The primary barrier was the lack of human resources—only one peer worker was hired for the housing site (unlike two peer workers being hired in Québec), and when he went on leave, there was nobody to replace him. Like other sites, implementation team meetings stopped early in the pandemic and there were many competing priorities. When asked about the implementation team’s current priorities, a tenant member of the team said:

Getting through the COVID crisis on a day to day basis as, you know, individuals and tenants at the Bell and in some cases friends, you know, and we’re not really speaking very much about the research project or you know peer support as well as the COVID lockdown and everything… Yes, basically everybody’s worries right now is, you know, getting through the lockdown with their sanity intact.

### Indefinite postponement with no decision on relaunch date

#### New Brunswick 1 (family support group)

This was the only rural site in this study and the only health service (rather than housing). At the time of the pandemic outbreak and ensuing lockdown policies, the first cohort of family members of someone living with mental health challenge had completed the 10-week family support group program and the second cohort had completed four of 10 weeks. In response to the COVID-19 ban on in-person gatherings, the family support group was stopped.

Though adapting to a virtual mode was looked upon favorably, they lacked the Internet, hardware (computers and webcams), and software (enough professional Zoom accounts), to make the shift, and were uncertain as to when they could start-up the group again. When asked what the priority of the implementation team was right now, the facilitator of the support group captured the intersection of the adaptability of the support group to a virtual mode, but also the challenges that access to resources would have:

Interviewee: Well on my side the priority would really be to reconnect and then discuss what is it going to be? Are we going to continue meetings then if we were only at the 3rd session of my group, to add people to make a new group of [name of program], to tie other people in need, then by making it virtual, I think it makes it easier.

Interviewer: Does that make it easier?

Interviewee: Yes.

Interviewer: Ok and how does it make it easier?

Interviewee: Well, I said it wrong, it doesn't make it easy, but it makes it easier to have different participants, so if we have someone who is seriously ill, it will certainly help from home, it gives the chance, the virtual, they can do it in their own living room, and that I think it helps a lot to approach people who cannot be present in person.

Interviewer: In person, travel and all that, the other challenge is to reach the people?

Interviewee: Yes, that's it. We stay in a very rural community where the internet is a little bit worse, and we are in a region where the finances are a little bit worse, so not everyone is lucky to have the internet and computers.

Interviewer: Computers.

Interviewee: That is going to be a little bit, a little of a challenge.

As implied above, the team had disconnected, as the relative priority of implementing the innovation fell by the wayside of other COVID-19-related priorities:

For sure that since COVID started, we haven’t had a committee [implementation team] meetings, it was really about responding to immediate needs that we took care of, and it wasn’t an immediate need, so we put it, we put it on the pile of things that we’ll have to do after…(Service provider, New Brunswick 1)

Although the relative priority of implementing the family support group was lower compared to dealing with the effects of COVID-19, the climate was not strongly negative, because members of the team believed there would be a greater receptivity and shared sense of need for the innovation. In the words of the family member on the implementation team:

Because personally I think that it will have really added pressure on families and then for people who are involved with the support of a family member who is mentally ill, there will be even more needs, I believe, to be able to have group discussions… (Family member, New Brunswick 1)

### No implementation of innovation yet

#### New Brunswick 2 (staff training)

This site was an outlier at this snapshot in time because it was the only site to have not yet started implementation of their innovation. The primary reason for the delay, pre-pandemic, was a lack of funding. However, just as the pandemic was hitting the province, funds had finally been secured (hence the positive influence of available resources) and plans were being made to contract the same trainer as the Ontario site to deliver a virtual training. COVID-19 had the impact of prolonging non-implementation as communication channels with the trainer had become slow. The personal attributes of the trainer at this moment in time (i.e., being unresponsive) was a strong negative influence (see Additional File 4). Priorities of the implementation team also shifted with the lead stretched too thin to be able to continue planning meetings while adjusting to externally driven COVID-19 policies.

I spent most of my time, in March and April, changing procedures just for work here, so the research project, it was really really really in the back on my [mind]. (Manager, New Brunswick 2)

This led to a 6-week pause in meetings. In the words of the tenant implementation team member “I think it’s [implementation of the innovation] in limbo probably mostly because of COVID”. However, unlike other sites that implemented staff training, adapting to an online format was not a barrier for this site because they had already grown accustomed to the idea and to the need to adapt to circumstances because of their funding challenges. When asked what made adapting hard or easy, a service provider put it this way:

I would say it’s pretty easy only because we had no choice, not having funding, we have no choice, we can’t afford the trainer that we wanted initially, we can’t afford to fly somebody here and do all that, but with regards to like the COVID-19, same thing, it’s been kind of easy just because we don’t have any other options…

In all, despite the less positive implementation outcome, a constellation of implementation facilitators was beginning to align for New Brunswick 2. Their biggest barriers were difficulty getting confirmation from the trainer, and the team dealing with many competing priorities.

## Discussion

The COVID-19 pandemic has, not surprisingly, had a significant impact on the implementation of recovery-oriented innovations into services in this project. This paper investigated the short-term impact (first two to three months of the pandemic). Longer-term impacts are expected but will be evaluated based on post-implementation data collection. In the mid-implementation phase of this study, only one site (Québec) was able to continue implementation in the early months of the pandemic outbreak (March–May 2020), with adaptation (going virtual versus in-person). Leadership engagement was a positive influence in all sites regardless of outcome. Leaders made a positive influence on implementation particularly by maintaining commitment to implementation despite COVID-19 and maintaining or making new resources available that facilitated adaptation. Our analysis highlights factors that appeared to be key drivers for continued adoption of innovations in the early months of the global pandemic for this site. Québec was the only site where the implementation team (formally appointed internal implementation leaders) did not postpone or cancel their meetings. This enabled planning and decision-making for an adapted innovation to continue. Available resources were also an important driver. Human resources were available in terms of the capacity of implementation team members to continue meeting and the fact that peer workers had been hired prior to the pandemic outbreak. Technological resources like Internet, computers, and tablets (both for team meetings, and for peer worker sessions) were also available.

Furthermore, the implementation climate and relative priority for a peer worker innovation was positive because peer support was seen as even more important in light of the impact the COVID-19 pandemic, and its associated policies, were having on tenants. During lockdown, service users may face a number of uncertainties which they may want an opportunity to discuss, such as experiencing social isolation, and being concerned about access to medications (42). During lockdown, many services deemed non-essential stopped, leaving service users without the social and skills support that are important to them (43).

Residents in supported housing had to live with greater restrictions than the general public. Labeled as a “vulnerable population” they were no longer allowed visitors, face-to-face support, lost access to certain services, and in Québec, residents were not allowed to leave their homes for essentials unless accompanied by the housing proprietor. When other restrictions were being lifted for society in general, persons living in supported housing were often the last to regain freedoms.

The immediate relative priority of staff training was low in comparison to more service user-aimed innovations (WRAP, peer workers, family support group). Staff had many competing priorities. Staff shortages, staff illness, and increased stress were important reasons to postpone until face-to-face was possible. For New Brunswick 2, the outlier in this research since they had not yet begun implementation, it was interesting that a number of facilitators to implementation commencement were aligning at the time of the COVID-19 pandemic (e.g., budget and finding a trainer). Adapting was New Brunswick 2’s *modus operandi* long before the COVID-19 outbreak. They had to adapt their training plans many times in the face of funding shortages. This experience had actually built capacity in the team to be open to virtual adaptations in a unique way compared to other sites which were less adaptive.

A significant driver of the choice to postpone until face-to-face activities could resume was the perception among implementation team members that the innovation was not qualitatively adaptable to an online format. For example, though technically adaptable, team members were concerned that online WRAP sessions would not provide an adequately supportive and safe environment, and that online staff training would be less effective than in-person training because staff would not be able to interact with each other in the same way. WRAP however has reportedly been delivered online in during the COVID-19 pandemic elsewhere [43]. Hasson-Ohayon and Lysaker [42] found that intersubjective connections can be maintained even after switching to online platforms. However, the implementation teams had many doubts about moving WRAP and training online.

Access to technology has been identified as a significant barrier for service users when shifting in-person services to online [42]. Lack of equity in access to technology is not only important for accessing services [66, 67], but also participating in implementation planning. Just as telemedicine may end up excluding vulnerable populations from access to services [67], so can online forms of working exclude service users in general from participating in implementation efforts. In this project, management was asked to commit to providing tenant members of the implementation team with smartphones or computers and access to Internet, a camera, microphone, and calling capacity when being recruited to join the study in 2017. This proved an extremely difficult objective to achieve, and at the time of the COVID-19 outbreak, tenant or service user members of the implementation team in at least four out of the seven sites did not have access to the technology they would have needed to continue online participation in planning meetings. Some staff also had inadequate Internet in their places of work. In this day and age, lack of access to information technology and Internet among tenants in supported housing, among health and social services staff, and among rural and economically disadvantaged populations stands to exacerbate existing inequalities. Reducing inequities vis-à-vis access to information technology should be a priority for governments and organizations across Canada and elsewhere.

### Strengths and limitations

Our assessment of the factors shaping implementation for each organization is based on the perspective of 3–5 key informants per site. Focusing on a small sample with information power [56] allowed us to balance the desire to empirically study what was happening in organizations, while at the same time not overburdening organizations and individuals at this turbulent time. However, had it been feasible, recruiting participants beyond the implementation team and providers of the innovation would have been interesting. Though we cannot know for sure what a different sampling approach, for example by saturation [68], might have made to the findings, one possibility is that with a larger sample ratings may have been slightly different, in particular because the condition for assigning a strong rating (+2 or −2) was that it be based on at least two participants’ interviews and explicit examples described [57]. The fact that our sample also included different stakeholder perspectives may have reduced the likelihood of the same idea and position being repeated, since different stakeholders may naturally see and relate to the innovation and organization differently. A larger sample, as well as longer interviews, could have helped collect data related to a larger number of constructs as well as enabled alternative quantitative approaches to exploring patterns in the data [69]. However, as qualitative researchers, we found the Damschroder and Lowery [57] method useful compared to other qualitative analysis approaches such as thematic analysis or conventional qualitative content analysis. The method offered us a way to take the data coded to the CFIR and go a step further, by summarizing the data, considering whether the influence of the construct was positive or negative and how much so, and in the end compare ratings and the qualitative data across sites to try to identify what constructs appeared to shape mid-implementation outcomes. The method balances the power of abstraction to explore higher level factors, while at the same time keeping the researcher close to the qualitative data.

A limitation in our application of the method is that coders could not be blinded to the outcomes as recommended by Damschroder and Lowery [57] because mid-implementation outcomes were made explicit in the transcripts themselves. This may have introduced researcher-bias in terms of interpreting the strength and valence of constructs; however, we believe this risk was mediated by a very rigorous approach of independent coding and rating by two researchers followed by consensus discussion. Our findings are also slightly weakened by the fact that we had seven sites and four implementation outcomes, meaning that three of the four outcomes were represented by only one site. Furthermore, we were looking at factors that shaped mid-implementation outcomes, a less studied set of outcomes than post-implementation outcomes. More methodological guidance on applying the Damschroder and Lowery [57] method to mid-implementation data could be useful for future research. Adding to the complexity was that in this study not all sites implemented the same innovation. However, this is not unprecedented and others have argued that although innovation diversity increases the complexity of an evaluation, the benefit is that it provides a wider range of intervention characteristics to evaluate [70].

Because we used CFIR as a data collection and analysis tool, adaptability was explored as a characteristic of the innovations and adapting as a mid-implementation outcome inseparable from continued adoption at this mid-implementation phase. A different but equally interesting way to approach mid-implementation evaluation might have been to simply focus on the adaptations made to innovations. As external facilitators, we did not introduce implementation teams to an adaptation framework that may have helped guide their adaptation decisions [41].

Lastly, this study has a relatively large number of interviewers (nine from seven sites) in relation to the number of interviews (27 in total). Since interviewers hone their skills as they get used to an interview guide, we might not have optimally benefitted from interviewer experience with the guide. However, all interviewers had experience with a CFIR-based interview guide from pre-implementation data collection. Nonetheless, we observed some heterogeneity in the richness and specificity of data for each construct in relation to interviewee style and skills (e.g., effective and on-topic prompting). We acted on this observation for post-implementation data collection by providing one-on-one coaching sessions with each interviewer to optimize data richness and consistency across sites.

## Conclusions

Our research highlights the negative impact that COVID-19, an outer setting factor, had on the implementation of recoveryinnovations into services. Due to most recovery innovations being predicated upon face-to-face interactions and relationship-building [71], lockdown policies made it impossible to continue implementation as planned. Only one of seven sites was able to continue implementing without interruption by adapting their innovation to a virtual format. However, it is also important to note that none of the sites outright canceled implementation, which itself is a very a positive outcome. In fact, every single site did resume implementation of adapted innovations eventually. So while it is important to study what factors shaped the immediate impact of COVID-19 early in the pandemic, like others have noted, some sites were able to overcome their barriers with a little more time [70]. The key factors which appeared to help sites continue implementation in the early months of the COVID-19 pandemic were continued engagement of the implementation team in planning, having the necessary resources to adapt and perceiving adaptations positively, and having a positive implementation climate vis-à-vis the specific innovation being implemented. Leadership support was a positive influence across all sites. It is possible that these factors are relevant to any extreme outer setting factor, such as a natural disaster, or serious economic crisis. It seems at the very least important to consider strategies that optimize these facilitating factors, such as external facilitation that support teams to continue their work, engaging leadership from the beginning to ensure continued commitment no matter what, and bolstering communications resources in organizations and among service users so that organizations can more easily adapt to disruptions that limit face-to-face interaction.

## Supplementary Information

**Additional file 1.** COREQ checklist

**Additional file 2.** StaRI checklist

**Additional file 3.** Interview Guide

**Additional file 4.** Ratings for all CFIR constructs by implementation outcome

**Additional file 5.** Ratings for all CFIR constructs by innovation

## Data Availability

The datasets used and/or analyzed during the current study are available from the corresponding author on reasonable request.

## Abbreviations

CFIR: Consolidated Framework for Implementation Research
COVID-19: Corona virus disease 2019
WRAP: Wellness Recovery Action Planning
