# Supplementary material for: Identifying and understanding the contextual factors that shaped mid-implementation outcomes during the COVID-19 pandemic in organizations implementing mental health recovery innovations into services
Source: Implement Sci Commun. 2021 Sep 15;2:101. doi: 10.1186/s43058-021-00206-w (PMC8441235; doi:10.1186/s43058-021-00206-w)
Supplement: Supplementary file 3 — Additional file 3. Interview Guide [file 43058_2021_206_MOESM3_ESM.docx]

Additional file to:

Piat et al. Identifying and understanding the contextual factors that shaped mid-implementation outcomes during the COVID-19 pandemic in organizations implementing mental health recovery innovations into services

**Mid-Implementation Individual Interview Guide**

**Members of the implementation team and other actors**

**Max. 45 minutes**

**INTRO:**

1. Let’s just start with a general question so we can understand the situation you are working/living in right now. What have been the big changes or shifts to the way your service/housing works since the outbreak of Covid19?

**OUTER SETTING:**

2. How has the Covid19 outbreak impacted on the implementation of [name of innovation] at [name of housing/service/organization]?

- Has the organization received specific policies or directives from government around COVID that have impacted on the implementation or roll-out of [name of innovation]. How have they impacted? [external policies and incentives]

**INTERVENTION CHARACTERISTICS:**

3. Have you had to adapt the [name of innovation], or have you thought about adapting it because of COVID? How? [Adaptability]

- How easy was it, or wasn’t it, to adapt the [name of innovation]?

- What made it easy or hard to adapt?

**INNER SETTING:**

4. Do you think COVID has or will have an impact on how receptive (interested in/open) people are to [name of the innovation] or how much support it will get? Why? How? [implementation climate]

*- [by “people” we mean service providers, or service users (families). Prompt them to think about both groups of people]*

- Have priorities shifted (for the organization, staff, service users, or families)? [relative priority]

- Have there been any shifts in leaders’ commitment to implement [name of innovation]? [leadership engagement]

5. Has there been an impact on resources, that is, people, time, or money for implementing the innovation? [available resources]

- If yes, what resources for [name of innovation] have been made available, or have been redirected, because of Covid?

**PROCESS:**

6. How has the outbreak of COVID affected the work of the implementation team? [formally-appointed internal implementation leaders]

- Have you been able to continue meeting remotely? What’s made this easy or hard?

- Have some members found it more difficult to continue engaging with the team than others? (because of quarantine, because of lack of access to internet or computer/phone, or because of other priorities at work or home)?

- What’s the implementation team’s priority right now?

7. Moving forward - how do you see the future for implementing [name of innovation]
