# Supplementary material for: Identifying and understanding the contextual factors that shaped mid-implementation outcomes during the COVID-19 pandemic in organizations implementing mental health recovery innovations into services
Source: Implement Sci Commun. 2021 Sep 15;2:101. doi: 10.1186/s43058-021-00206-w (PMC8441235; doi:10.1186/s43058-021-00206-w)
Supplement: Supplementary file 4 — Additional file 4. Ratings for all CFIR constructs by implementation outcome [file 43058_2021_206_MOESM4_ESM.docx]

**Table 3. Ratings assigned to CFIR constructs by site and mid-implementation outcome**

| **Site, Funding, Innovation** | **New Brunswick 2**  **Not-for-Profit, Staff Training** | **New Brunswick 1, publicly funded, family support group** | **British Columbia, not-for-profit, WRAP** | **Manitoba 1 , not-for-profit, Staff Training** | **Manitoba 2**  **not-for-profit, Peer Worker** | **Ontario**  **not-for-profit, Staff Training** | **Quebec,**  **Publicly funded, Peer Workers** |
| --- | --- | --- | --- | --- | --- | --- | --- |
| **Mid-Implementation outcome**  **CFIR Domains and Constructs** | **No implementation of innovation yet** | **Indefinite postponement with no decision on relaunch date** | **Postponement with adaptation and estimated relaunch date** | | | | **Continued implementation with adaptation** |
| **I. INNOVATION CHARACTERISTICS** |  |  |  |  |  |  |  |
| A. Innovation Source |  | +1 |  |  |  |  |  |
| B. Evidence Strength and Quality |  |  |  |  |  |  |  |
| C. Relative Advantage | +1 |  | +1 |  | +1 |  |  |
| **D. Adaptability** | **+2** | +1* | **-2** | **+2** | -1 | **-2** | +1* |
| E. Trialability |  |  |  |  |  |  |  |
| F. Complexity |  |  |  |  |  |  |  |
| G. Design Quality & Packaging | +1 |  |  |  | +1 | +1 | **+2** |
| H. Cost | **+2** |  | +1 |  | -1 |  |  |
| **II. OUTER SETTING** |  |  |  |  |  |  |  |
| A. Needs & Resources of Those Served by the Organization |  | **+2** | 0 | 0 | 0 | -1 | +1 |
| B. Cosmopolitanism |  | **+2** |  |  |  |  |  |
| C. Peer Pressure | +1 |  |  |  |  |  |  |
| **D. COVID-19-related external policy** | -1 | **-2** | **-2** | **-2** | **-2** | **-2** | **-2** |
| E. Local severity of the COVID-19 pandemic and quality of response | +1 | 0 | +1 | 0 | +1 | 0 | 0 |
| F. Priority given to mental health in wider society | -1 |  |  |  |  | 0 |  |
| **III. INNER SETTING** |  |  |  |  |  |  |  |
| A. Structural Characteristics | -1 |  |  |  | -1 |  | 0 |
| B. Networks & Communications |  |  | -1 | 0 |  |  | 0 |
| C. Culture | -1 | 0 |  | +1 | 0 | **-2** | 0 |
| **D. Implementation Climate and Relative Priority** | **-2** | -1 | -1* | +1* | -1 | **-2*** | **+2*** |
| d1. Tension for Change |  |  |  |  |  |  | +1. |
| d2. Compatibility |  | +1 |  | +1 | +1 |  | 0 |
| d4. Organizational Incentives and Rewards |  |  |  |  |  |  |  |
| d5. Goals and Feedback |  |  |  |  |  |  |  |
| d6. Learning Climate |  |  |  |  | -1 | -1 | +1* |
| E. Readiness for Implementation |  | +1 | +1 | 0 |  |  |  |
| **e1. Leadership Engagement** | +1 | -1 | **+2** | **+2** | +1 | +1* | **+2** |
| **e2. Available Resources** | +1* | **-2** | -1* | -1 | -1 | +1 | **+2*** |
| e3. Access to Knowledge & Information |  |  |  | -1 |  |  | +1 |
| **IV. CHARACTERISTICS OF INDIVIDUALS** |  |  |  |  |  |  |  |
| A. Knowledge & Beliefs about the Innovation |  | +1 | +1 |  |  | 0 |  |
| B. Self-Efficacy |  |  |  |  |  |  |  |
| C. Individual Stage of Change |  |  |  |  |  |  |  |
| D. Individual Identification with Organization | +1 |  |  |  | +1 |  |  |
| E. Other Personal Attributes | **-2** | +1 | **-2** | -1 | -1 | +1 | +1 |
| **V. PROCESS** |  |  |  |  |  |  |  |
| A. Planning |  |  |  |  |  |  | +1 |
| B. Engaging |  |  |  |  |  |  |  |
| b1. Opinion Leaders |  |  |  |  |  | +1 |  |
| **b.2 Engaging implementation teams during the COVID-19 pandemic** | **-2** | **-2** | -1 | -1 | -2 | **-2** | **+2** |
| b3. Champions | -1 |  | 0 |  |  | +1 |  |
| b4. External Change Agents | **+2** | +1 | **+2** | 0 | 0 | +1 | +1 |
| b5. Key Stakeholders | **-2** |  |  | +1 | 0 | +1 | +1 |
| b6. Innovation Participants | 0 | +1 |  | +1 | -1 |  | +1 |
| C. Executing |  |  |  |  |  |  |  |
| D. Reflecting & Evaluating |  |  |  |  |  |  | +1 |

CFIR Constructs that are **bolded and underlined** are those that were explicitly targeted by a question in the interview guide. * indicates that there exists a view that is contrary to the overall rating. Consolidated Framework for Implementation Research (CFIR).
