# Supplementary material for: Identifying and understanding the contextual factors that shaped mid-implementation outcomes during the COVID-19 pandemic in organizations implementing mental health recovery innovations into services
Source: Implement Sci Commun. 2021 Sep 15;2:101. doi: 10.1186/s43058-021-00206-w (PMC8441235; doi:10.1186/s43058-021-00206-w)
Supplement: Supplementary file 5 — Additional file 5. Ratings for all CFIR constructs by innovation [file 43058_2021_206_MOESM5_ESM.docx]

**Table 3. Ratings assigned to CFIR constructs by site and innovation being implemented**

| Site | New Brunswick 1 | British Columbia | New Brunswick 2 | Ontario | Manitoba 2 | Manitoba 1 | Québec |
| --- | --- | --- | --- | --- | --- | --- | --- |
| Innovation  CFIR Domains and  constructs | **Family support group** | **WRAP** | **Staff training** | | | **Peer Workers** | |
| **I. INTERVENTION CHARACTERISTICS** |  |  |  |  |  |  |  |
| A. Innovation Source | +1 |  |  |  |  |  |  |
| B. Evidence Strength and Quality |  |  |  |  |  |  |  |
| C. Relative Advantage |  | +1 | +1 |  | +1 |  |  |
| **D. Adaptability** | +1* | **-2** | **+2** | **-2** | -1 | **+2** | +1* |
| E. Trialability |  |  |  |  |  |  |  |
| F. Complexity |  |  |  |  |  |  |  |
| G. Design Quality & Packaging |  |  | +1 | +1 | +1 |  | **+2** |
| H. Cost |  | +1 | **+2** |  | -1 |  |  |
| **II. OUTER SETTING** |  |  |  |  |  |  |  |
| A. Needs & Resources of Those Served by the Organization | **+2** | 0 |  | -1 | 0 | 0 | +1 |
| B. Cosmopolitanism | **+2** |  |  |  |  |  |  |
| C. Peer Pressure |  |  | +1 |  |  |  |  |
| **D. COVID-19-related external policy** | **-2** | **-2** | -1 | **-2** | **-2** | **-2** | **-2** |
| E. Local severity of the COVID-19 pandemic and quality of response | 0 | +1 | +1 | 0 | +1 | 0 | 0 |
| F. Priority given to mental health in wider society |  |  | -1 | 0 |  |  |  |
| **III. INNER SETTING** |  |  |  |  |  |  |  |
| A. Structural Characteristics |  |  | -1 |  | -1 |  | 0 |
| B. Networks & Communications |  | -1 |  |  |  | 0 | 0 |
| C. Culture | 0 |  | -1 | **-2** | 0 | +1 | 0 |
| **D. Implementation Climate and Relative Priority** | -1 | -1* | **-2** | **-2*** | -1 | +1* | **+2*** |
| d1. Tension for Change |  |  |  |  |  |  | +1. |
| d2. Compatibility | +1 |  |  |  | +1 | +1 | 0 |
| d3. Relative Priority |  |  |  |  |  |  |  |
| d4. Organizational Incentives and Rewards |  |  |  |  |  |  |  |
| d5. Goals and Feedback |  |  |  |  |  |  |  |
| d6. Learning Climate |  |  |  | -1 | -1 |  | +1* |
| dE. Readiness for Implementation | +1 | +1 |  |  |  | 0 |  |
| **d1. Leadership Engagement** | -1 | **+2** | +1 | +1* | +1 | **+2** | **+2** |
| **d2. Available Resources** | **-2** | -1* | +1* | +1 | -1 | -1 | **+2*** |
| d3. Access to Knowledge & Information |  |  |  |  |  | -1 | +1 |
| **IV. CHARACTERISTICS OF INDIVIDUALS** |  |  |  |  |  |  |  |
| A. Knowledge & Beliefs about the Innovation | +1 | +1 |  | 0 |  |  |  |
| B. Self-Efficacy |  |  |  |  |  |  |  |
| C. Individual Stage of Change |  |  |  |  |  |  |  |
| D. Individual Identification with Organization |  |  | +1 |  | +1 |  |  |
| E. Other Personal Attributes | +1 | **-2** | **-2** | +1 | -1 | -1 | +1 |
| **V. PROCESS** |  |  |  |  |  |  |  |
| A. Planning |  |  |  |  |  |  | +1 |
| B. Engaging |  |  |  |  |  |  |  |
| b1. Opinion Leaders |  |  |  | +1 |  |  |  |
| **b2. E****ngaging implementation teams during the COVID-19 pandemic** | **-2** | -1 | **-2** | **-2** | -2 | -1 | **+2** |
| b3. Champions |  | 0 | -1 | +1 |  |  |  |
| b4. External Change Agents | +1 | **+2** | **+2** | +1 | 0 | 0 | +1 |
| b5. Key Stakeholders |  |  | **-2** | +1 | 0 | +1 | +1 |
| b6. Innovation Participants | +1 |  | 0 |  | -1 | +1 | +1 |
| C. Executing |  |  |  |  |  |  |  |
| D. Reflecting & Evaluating |  |  |  |  |  |  | +1 |

CFIR Constructs that are **bolded and underlined** are those that were explicitly targeted by a question in the interview guide. * indicates that there exists a view that is contrary to the overall rating. Consolidated Framework for Implementation Research (CFIR), Wellness Recovery Action Planning (WRAP). * indicates that there exists a view that is contrary to the overall rating.
